# Bud structure, position and fate generate various branching patterns along shoots of closely related Rosaceae species: a review

**DOI:** 10.3389/fpls.2014.00666

**Published:** 2014-12-02

**Authors:** Evelyne Costes, Laurent Crespel, Béatrice Denoyes, Philippe Morel, Marie-Noëlle Demene, Pierre-Eric Lauri, Bénédicte Wenden

**Affiliations:** ^1^INRA, Unité Mixte de Recherche 1334, Amélioration Génétique et Adaptation des Plantes Méditerranéennes et Tropicales Centre de Coopération Internationale en Recherche Agronomique pour le Développement-INRA-Montpellier SupAgro, Architecture et Fonctionnement des Espèces Fruitières TeamMontpellier, France; ^2^Agrocampus Ouest, Institut de Recherche en Horticulture et Semences INRA-Agro Campus Ouest-Université d'AngersAngers, France; ^3^INRA, Unité Mixte de Recherche 1332, Biologie du Fruit et Pathologie, Université de Bordeaux-INRAVillenave d'Ornon, France; ^4^INRA, Institut de Recherche en Horticulture et Semences, INRA-Agro Campus Ouest-Université d'AngersBeaucouzé, France; ^5^InvenioDouville, France

**Keywords:** plant architecture, SAM determinacy, SAM identity, flowering, vegetative development, growth phases

## Abstract

Branching in temperate plants is closely linked to bud fates, either floral or vegetative. Here, we review how the fate of meristematic tissues contained in buds and their position along a shoot imprint specific branching patterns which differ among species. Through examples chosen in closely related species in different genera of the Rosaceae family, a panorama of patterns is apparent. Patterns depend on whether vegetative and floral buds are borne individually or together in mixed buds, develop as the shoot grows or after a rest period, and are located in axillary or terminal positions along the parent shoot. The resulting branching patterns are conserved among varieties in a given species but progressively change with the parent shoot length during plant ontogeny. They can also be modulated by agronomic and environmental conditions. The existence of various organizations in the topology and fate of meristematic tissues and their appendages in closely related species questions the between-species conservation of physiological and molecular mechanisms leading to bud outgrowth vs. quiescence and to floral induction vs. vegetative development.

## Introduction

Polycarpic plants are characterized by the co-existence of multiple axes which result from the activity of different meristems. These axes can be similar or morphologically differentiated. In temperate species, axes have been classified depending on the presence vs. absence of neoformed organs, i.e., organs that were not included in the bud at an embryonic stage but formed during the morphogenesis and elongation period of the shoot (see also glossary in Supplementary Material). Typically, short axes are composed of preformed organs only whereas long shoots are composed of preformed organs followed by neoformed ones (see Costes et al., 2006). Another category corresponds to epicormic axes that are assumed to be entirely neoformed and develop in stressful conditions or after a severe pruning (Nicolini et al., 2003; Negron et al., 2014). At the whole plant scale, architecture and branching result from the relationships between the different buds and meristematic tissues that constitute them. Buds are located either terminally or axillary along the shoots. These relationships dictate how each bud develops, grows, or stops growing, i.e., whether the corresponding meristem maintains its organogenetic activity (i.e., its capability to generate new organs) or differentiates into specific organs such as thorns, flowers or inflorescences. Plants exhibit a limited number of possible branching configurations which make them conform to 22 architectural models (Hallé et al., 1978). Each architectural model corresponds to a particular combination of four main criteria that are related to the temporal and topological positioning of flowering and vegetative growth. One criterion concerns branching which can be immediate or delayed, monopodial, or sympodial with basitonic, mesotonic, or acrotonic positioning (see Barthélémy and Caraglio, 2007 and glossary). Moreover, sequential branching has been distinguished from reiteration that occurs when a lateral shoot has a comparable or longer length than its parent shoot and partially or totally repeats the parental branching system (Oldeman, 1974; Bell, 1991). Based on these founder studies, branching has been described and sometimes quantified in a number of different forest and fruit tree species (e.g., Suzuki, 2002; Renton et al., 2006; Solar and Stampar, 2006).

In parallel, the physiological and genetic mechanisms underlying meristem organogenesis and the control of axillary meristem outgrowth have been extensively studied. Apical dominance is considered to be a function of auxin (IAA) production in the apical meristem (Thiman and Skoog, 1933 in Cline, 2000). The screening of mutants, mainly in annual plants such as *Arabidopsis thaliana* (Leyser, 2003), rice (Li et al., 2003) and pea (Beveridge et al., 2000; Foo et al., 2001), has led to considerable improvement of knowledge in these domains. Apical dominance has been shown to involve auxin transport down the shoot via an active transporter in the parenchyma associated with xylem tissue (Booker et al., 2003). However, when axillary meristems being controlled are distant from the apex, the speed of IAA transport appears incompatible with its putative role in axillary bud inhibition (Renton et al., 2012). The formation of axillary meristems has been shown to require a Lateral suppressor gene which is expressed in the boundary region between the leaf primordium and stem (*Ls* in tomato, Schumacher et al., 1999; or *LAS* in *A. thaliana*, Greb et al., 2003 and rice, Li et al., 2003). Moreover, lateral branching has been shown to be under the control of a complex interaction between cytokinins (CK) that are promoters of bud outgrowth, auxin (IAA) as a repressor, but also strigolactones (SL) (Leyser, 2009), that inhibit the axillary bud outgrowth (Gomez-Roldan et al., 2008; Umehara et al., 2008). The current debate on the hormonal control of branching focuses on the interactions of auxin with SL and CK. Some studies suggest that SLs act directly in the bud to inhibit bud outgrowth, thus acting as secondary messenger to auxin (Brewer et al., 2009; Dun et al., 2013). Another hypothesis proposes that SLs impede the ability of buds to export auxin into the main stem, and hence inhibit their outgrowth (Domagalska and Leyser, 2011). The *BRANCHED 1* (*BRC1*) gene likely integrates these multiple bud outgrowth pathways (Braun et al., 2012). However, a recent study revealed that the shoot tip's strong demand for sugars, rather than auxin supply, inhibits axillary bud outgrowth by limiting the amount of sugar translocated to those buds (Mason et al., 2014).

In the present paper, we aim at extending the current debate to more complex plants with different timings and locations of meristem outgrowths. In particular, polycarpic perennial plants growing in temperate conditions are characterized by the formation of buds that are able to survive winter periods and resume growth at spring. Moreover, these plants can be viewed as a population of meristems, each of them having different stages of differentiation and passing from one stage to the next one through remarkable transitions during ontogeny (White, 1979). However, these different meristems are linked together by the plant age, resource availability, and environmental conditions in which the plant develops. Recent studies have outlined close relationships between molecular control of shoot apical meristem transitions, especially floral transitions, entrance/release of dormancy, and the control of axillary meristem branching (Paul et al., 2014; van der Schoot et al., 2014).

We review branching organization in temperate Rosaceae species used for ornament or fruit production. The Rosaceae family constitutes an interesting case study since it includes many economically important species but also contains very diverse plant forms; trees (e.g., *Prunus* and *Malus*), bushes, and lianas, (e.g., *Rosa*), and herbaceous rosettes (e.g., *Fragaria*). Through examples chosen in closely related species from different genera of the Rosaceae family, a panorama of branching patterns is apparent. The patterns depend on whether lateral shoots develop immediately or after a rest period, flowering occurs in axillary or terminal positions and in singular or mixed buds. This reveals contrasting topological arrangements of vegetative and floral tissues that can be:

- Separated in individual axillary buds located in distinct zones in a tall tree, with strong apical dominance and acrotony (cherry),
- Separated in axillary buds that are distinct but can be produced together at the same node in trees, with apical dominance and acrotony (almond), a tendency toward basitony (peach), or a tendency toward sympodial branching,
- Combined into mixed buds located in terminal positions, composed of leaf primordia and a terminal inflorescence, that develops after a vegetative period lasting one to several years (apple), 1 year (“non-recurrent” or “once flowering,” rose and strawberry), several weeks (“recurrent” or “perpetual flowering” rose and strawberry, respectively).

The different configurations in the topology and fate of meristematic tissues and their appendages described in closely related species questions the evolution within the Rosaceae family and the co-adaptation of plant forms with their environment. They also question the between-species conservation of physiological and molecular mechanisms leading to bud outgrowth vs. quiescence, and to floral induction vs. vegetative development.

## Comparative description of branching patterns in rosaceae

### Trees with vegetative and floral tissues separated in axillary buds located in distinct zones

#### With apical dominace, acrotony and monopodial branching: the peach and almond case

**Figure 1:**
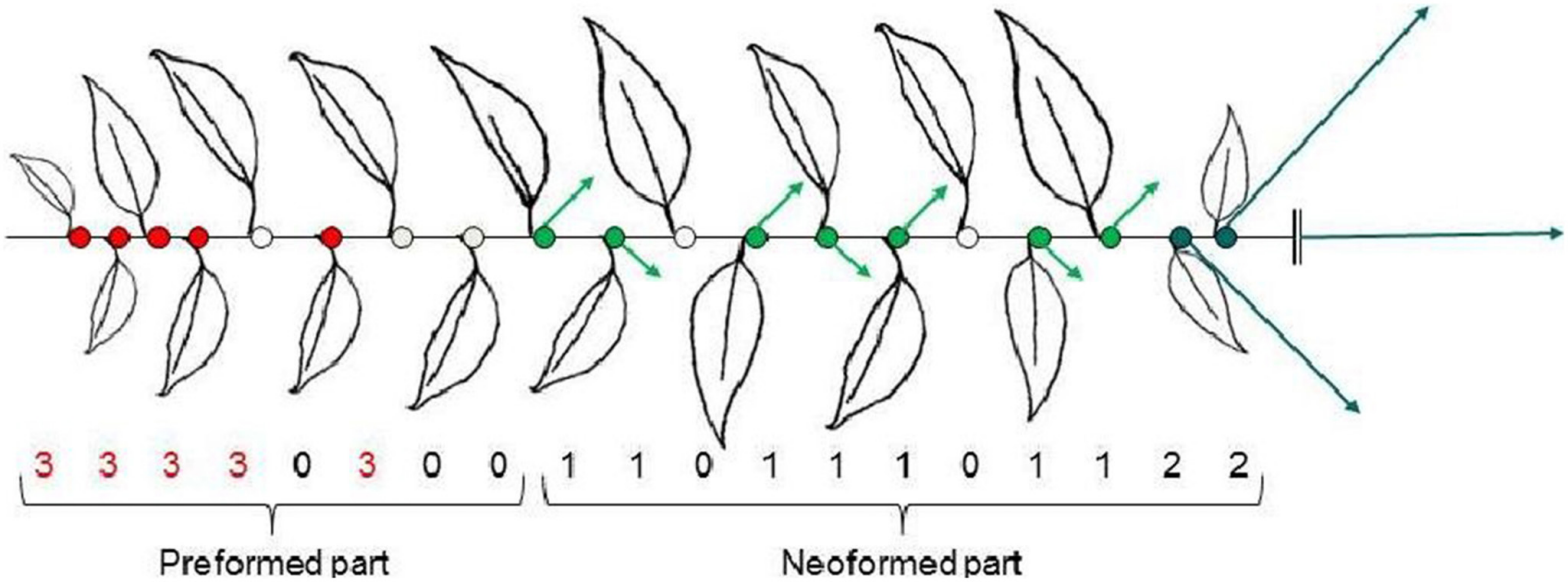
**Branching pattern along a cherry annual shoot—Flower buds are located along the preformed part and vegetative buds along the neoformed part**. 0 latent bud, 1 short proleptic shoot, 2 long proleptic shoot, 3 floral bud. Arrows indicate proleptic shoot lengths to be developed in the year *n* + 1.

**In the cherry tree** (*Prunus avium*), growth and branching are strictly monopodial at all stages of growth. Trees have a strong dimorphism between short or long shoots, composed of preformed only or preformed and neoformed organs, respectively (see glossary). The framework of the tree is composed of the trunk, with long and upright side-branches that usually develop with a strong acrotony, forming tiers of branches just below the annual growth arrest of the bearing shoot. These traits conform to the Rauh model of Hallé et al. (1978). The long branches bear short shoots, also called spurs, in lateral positions on the distal half or two thirds of the branch with more vigorous spurs toward the distal part (Lauri, 1993). Flowering occurs in axillary positions on the five to six basal-most nodes of all shoots whether long or short (Flore and Layne, 1996). Floral buds are thus located exclusively on the preformed nodes of the previous year shoots. Flowers are groups of two to five in umbels and are initiated within the buds formed in the leaf axils in the growing season prior to anthesis (Guimond et al., 1998). These flower buds will burst in the following year leaving bare wood after fruit harvest on the proximal part of long shoots, and on all nodes of the annual growth increment of the spurs. As a consequence, the typical branching pattern on the 1-year-old branch consists in an initial basal zone with single axillary buds containing inflorescences directly, followed by a zone of spurs and a top tier of long lateral shoots (Figure 1). One-year-old shoots, whether long or short, bear flowers in their basal part, over consecutive years. This basic branching pattern thus corresponds to a clear separation between zones along vegetative axes and lateral flowering. It varies depending on genotype, with sour cherry having more flower buds on long shoots than sweet cherries (Thompson, 1996).

### Trees with vegetative and floral tissues separated in distinct axillary buds, produced together at a same node

#### With apical dominance, acrotony, and monopodial branching: the almond and peach case

**Almond and peach trees** also have a monopodial and acrotonic branching at all growth stages. They conform to the Rauh architectural model (Fournier, 1994). In almond, laterals typically develop at nodes located one-half to two-thirds the distance from the tip of annual shoots, with a strong shoot dimorphism (Gradziel, 2009). Peach trees tend to be basitonic when trees are young, this species being typically bushy in its natural habit (Lauri, 1991). The bushy habit is related to a high frequency of sylleptic branching on the proximal part of the main shoot, the appearance of sylleptic laterals being positively related to the leaf emergence rate of the growing shoot (Génard et al., 1994). However, a typical acrotony is observed at the shoot level with, as in almond, a strong shoot dimorphism.

**Figure 2:**
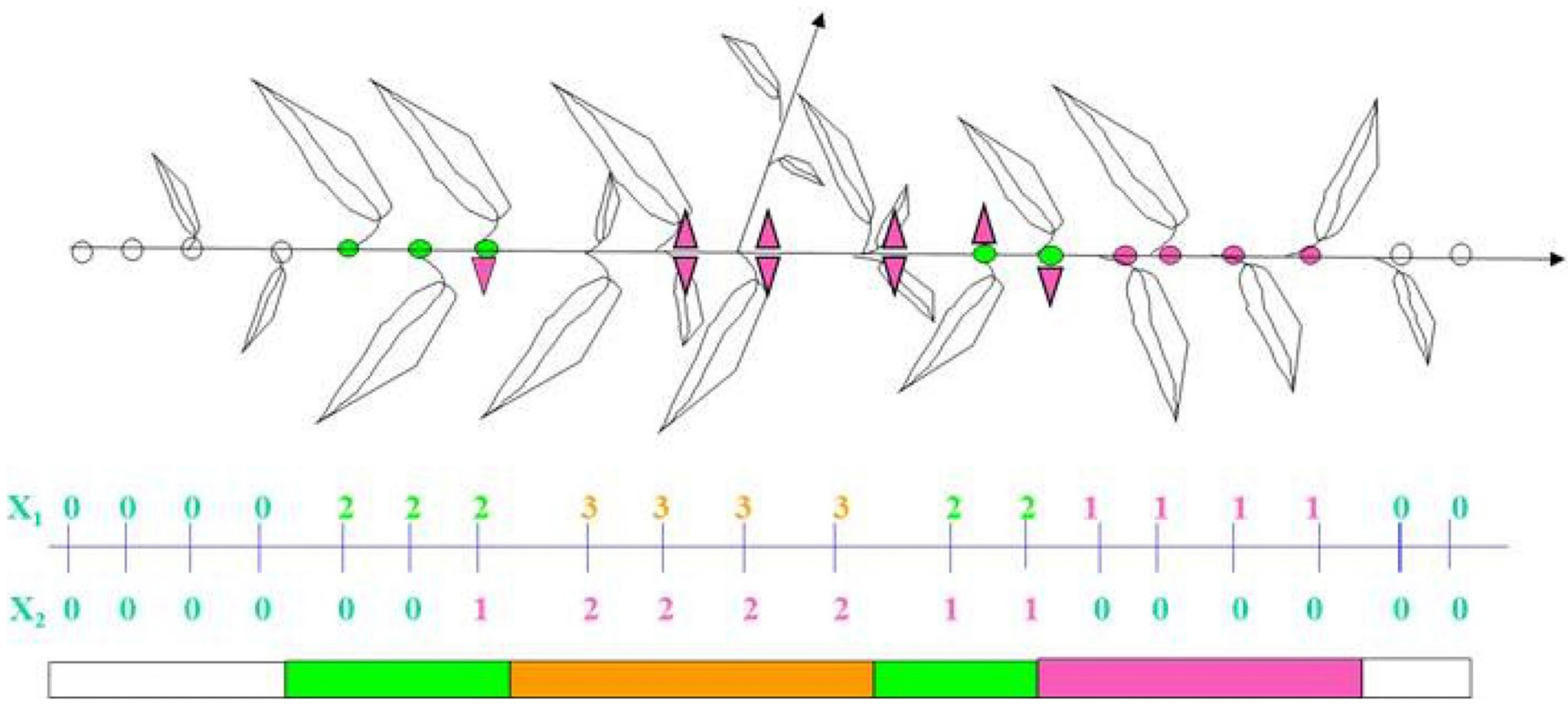
**Branching pattern along a peach annual shoot, “Robin” cultivar**. First variable: 0 latent bud, 1 isolated floral bud, 2 vegetative bud, 3 sylleptic shoot. Second variable: number (0, 1, 2) of axillary flowers and schematic representation of the shoot as a succession of zones (From Fournier et al., 1998). The segmentation of the parent shoot in branching zones with distinct combinations of vegetative and floral axillary buds is indicated in the lower part of the Figure.

In both species, axillary buds can be vegetative, floral, or blind. The floral buds enclose a single, terminal flower, and typical of *Prunus* species, no leaves. Along a parent shoot, flowers can be directly inserted on the parent shoot or on the first scales of the axillary vegetative buds. In these later cases, they can be grouped by two or more and appear borne laterally in leaf axils on parent shoots, either long or short (Lamp et al., 2001). In both almond and peach, branching organization along a 1-year old shoot can be described by combining two qualitative variables which take into account (i) the fate of the axillary meristem (latent, floral, vegetative, or sylleptic) and (ii) the number of axillary flowers (0, 1, 2, or more) associated with the axillary vegetative buds (Fournier, 1994). In both peach and almond, a modeling approach based on Markov chains (Guédon et al., 2001), revealed an organization of lateral bud fates in consecutive zones which follow each other in an almost deterministic way (Fournier et al., 1998; Negron et al., 2013). In “Nonpareil,” the main almond scion cultivar in California, and in “Robin,” a white peach scion cultivar, the typical branching pattern along 1-year-old long proleptic shoots has been described as a succession of six zones, each one defined according to its composition of axillary meristem fates and number of flowers (Figure 2). Blind nodes were observed at the proximal and distal ends of the shoots. Zones with vegetative buds mainly were associated with few flower buds whereas a zone with a mixture of sylleptic shoots and vegetative buds was associated with many flower buds. Also central floral buds were observed in a zone located in the top third to the shoot, below the terminal bud.

#### With tendency to shoot apical meristem death and sympodial branching: the apricot case

**In the apricot tree** (*Prunus armeniaca*), shoots are characterized by sympodial branching resulting from the frequent abortion of shoot apical meristem at growth cessation (Costes, 1993). This death can occur even during the growing season, thus separating the annual shoot into growth units (see glossary). In young trees, the growth units are usually long, and their branching pattern has been characterized by a short zone with latent buds at the base followed by numerous short axillary shoots, mixed with latent buds and few long axillary shoots. The position of the long axillary shoots is mainly acrotonic but also depends on the orientation of the parent shoot (Wareing and Nasr, 1958). Frequent bending of the shoots and the consequent gravimorphic reaction leads the tree to grow according to a Champagnat model (Costes, 1993). The flowers are, as in peach and almond, contained in single flower buds and associated at the axil of main vegetative buds along the parent shoot. The branching structure along growth units of “Lambertin” apricot scion cultivar has been quantified as a succession of zones differentiated by the type of laterals and the number of flowers with several recurrent zones (Costes and Guédon, 1996). The basal part of the growth units contains two zones, the first one with latent buds and no flowers, the second one with short vegetative laterals without flowers. Then five zones occur recurrently: a zone with latent buds and no flowers; a zone with sylleptic laterals; the last three zones with different numbers of flowers (1, 2, and more than 2) observed in succession. The number of flowers increases from 1 to 2 and 3, but never directly from 1 to 3 (or decreasing similarly). This suggests that flowering in axillary buds fluctuates in intensity depending on the bud position and timing of differentiation along the parent shoot.

### Vegetative and floral tissues combined in a mixed bud, located in terminal position

#### Tree with sympodial branching and acrotony: the apple case

**Figure 3:**
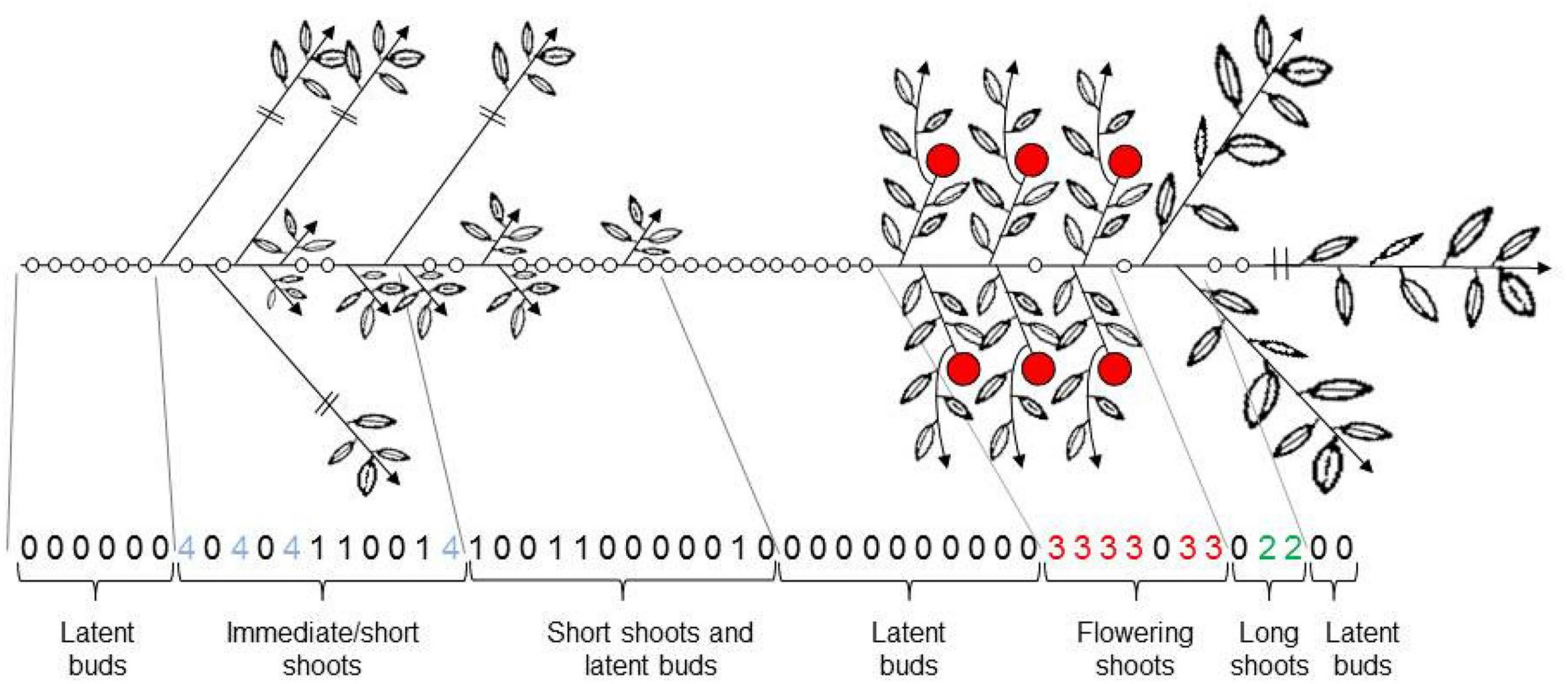
**Branching pattern along an apple long annual shoot**. The segmentation of the parent shoot in branching zones with a distinct dominant axillary shoot type is indicated. 0 latent bud, 1 short proleptic shoot, 2 long proleptic shoot, 3 floral shoot, 4 sylleptic shoot (From Guédon et al., 2001).

**In the apple tree**, branching is monopodial before the occurrence of flowering and lateral shoots are displayed according to an acrotonic gradient (Crabbé, 1987; Lauri, 2007). Thus, during the juvenile phase or during the vegetative state of non-flowering scions, the apple tree develops according to a Rauh model (Lauri and Térouanne, 1995). However, because floral differentiation occurs in terminal positions on all axes, the monopodial growth phase ends after flowering. The winter floral bud is constituted of a leafy basal part followed by a floral distal part (Fulford, 1966a,b; Abbott, 1984) and thus corresponds to a mixed bud. The mixed bud includes preformed leaf primordia of the lateral shoot that will continue the axis growth through sylleptic and sympodial branching (Barnola and Crabbé, 1991). The axillary shoot borne on the floral unit is called a “bourse shoot.” After the winter period, some of the axillary meristems develop and, as a consequence of acrotony, those located just below the terminal bud develop into long axillary shoots. Floral buds located at specific positions along the parent shoot, below this acrotonic zone (Costes and Guédon, 2002). The other axillary meristems can remain latent or develop into spurs. Some axillary meristems along the parent shoot may develop immediately, i.e., during the growth of a parent shoot. In apple trees, as in other species, such sylleptic shoots develop mainly during the early years of tree life (Crabbé, 1987), and in median position along long to very long parent shoots. As a whole, long parent shoots have been characterized by a zonation of branching, corresponding to successive zones that differentiate from each other by the relative proportion of latent, vegetative, and flowering buds. Depending on the cultivar, 6–7 zones have be distinguished along a long parent shoot (Guédon et al., 2001; Costes and Guédon, 2002; Figure 3).

#### Bush to liana with sympodial branching, basitony at the plant level and acrotony at the shoot level: the rose case

**Figure 4:**
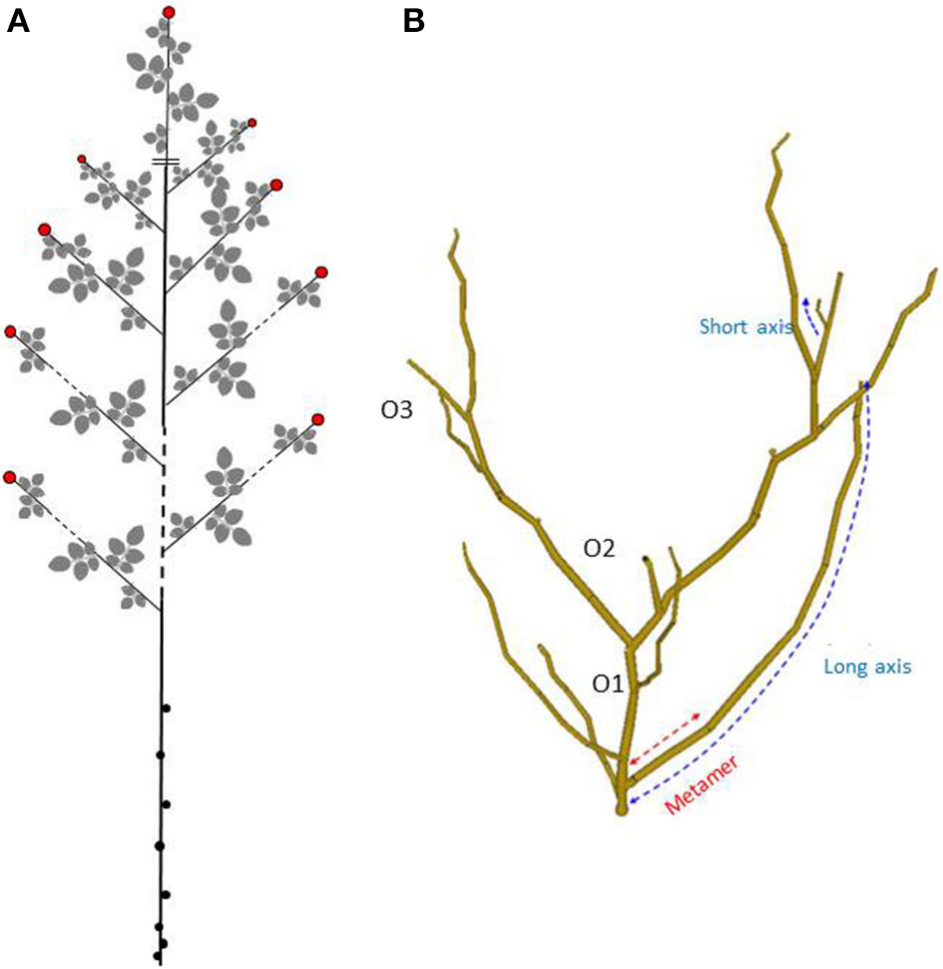
**(A)** Representation of a n-1 year old axis carrying n year old floriferous axes (terminal flower in red) in a non-recurrent flowering rose; **(B)** Representation obtained by digitizing of an elementary architectural structure of a rose having an upright growth habit, aged from 5 to 6 months, cultivated in greenhouse. Three architectural components (metamer, short, and long axes) and three branching orders are labeled (O1, O2, and O3) (From Crespel et al., 2013).

***Rosa*** plants develop according to the model of Champagnat (Le Bris, 1999). They are characterized by defined growth due to terminal flowering and a subsequent sympodial branching in all axes. The mode of floral induction makes it possible to distinguish non recurrent-flowering from recurrent-flowering behaviors (Roberts and Blake, 2003). For non-recurrent flowering roses, floral induction is dependent on the environmental conditions in particular winter cold under natural conditions (Roberts and Blake, 2003). Under these conditions, axes develop from the terminal and axillary buds of the previous year growth, mainly in distal positions and according to an acrotonic gradient. Some of these shoots will flower terminally (only once a year), leading to determinate growth (Figure 4A), others staying vegetative until the next year. These floriferous axes are much shorter than their parent axis.

For recurrent-flowering roses which represent the majority of cultivated roses, the flowering is auto-inducible and systematic, i.e., not subject to environmental conditions, provided that a trophic minimum is reached, and usually corresponding to production of six to seven leaves (Roberts and Blake, 2003). Under these conditions, during the growth period, all developing axes become floriferous. Branching after flowering is acrotonic and often sylleptic (Le Bris, 1999). Due to recurrent flowering, the number of successive sympodial branching orders can reach five after 5–6 months. The axis length reduces with consecutive branching orders, the long axes being located mainly on the first two orders, whereas the short axes are located at higher orders (Morel et al., 2009; Crespel et al., 2013). This branching organization and types of axes characterize the elementary architectural structure of rose bushes (Figure 4B). The axes in the acrotonic part exhibit dimorphism and their characteristics depend on the genotype (Crespel et al., 2014). In addition, other axillary shoots may develop in basitonic locations, from proximal buds which correspond to collateral or axillary buds located at the basal scales of axes (Marcelis-van Acker, 1993; Morel et al., 2009). The development of this type of axillary shoot can be viewed as a reiteration since it will lead to the repetition of the elementary architectural structure.

#### Herbaceous rosette with sympodial branching, basitony at the plant level and acrotony at the rosette level: the strawberry case

**Figure 5:**
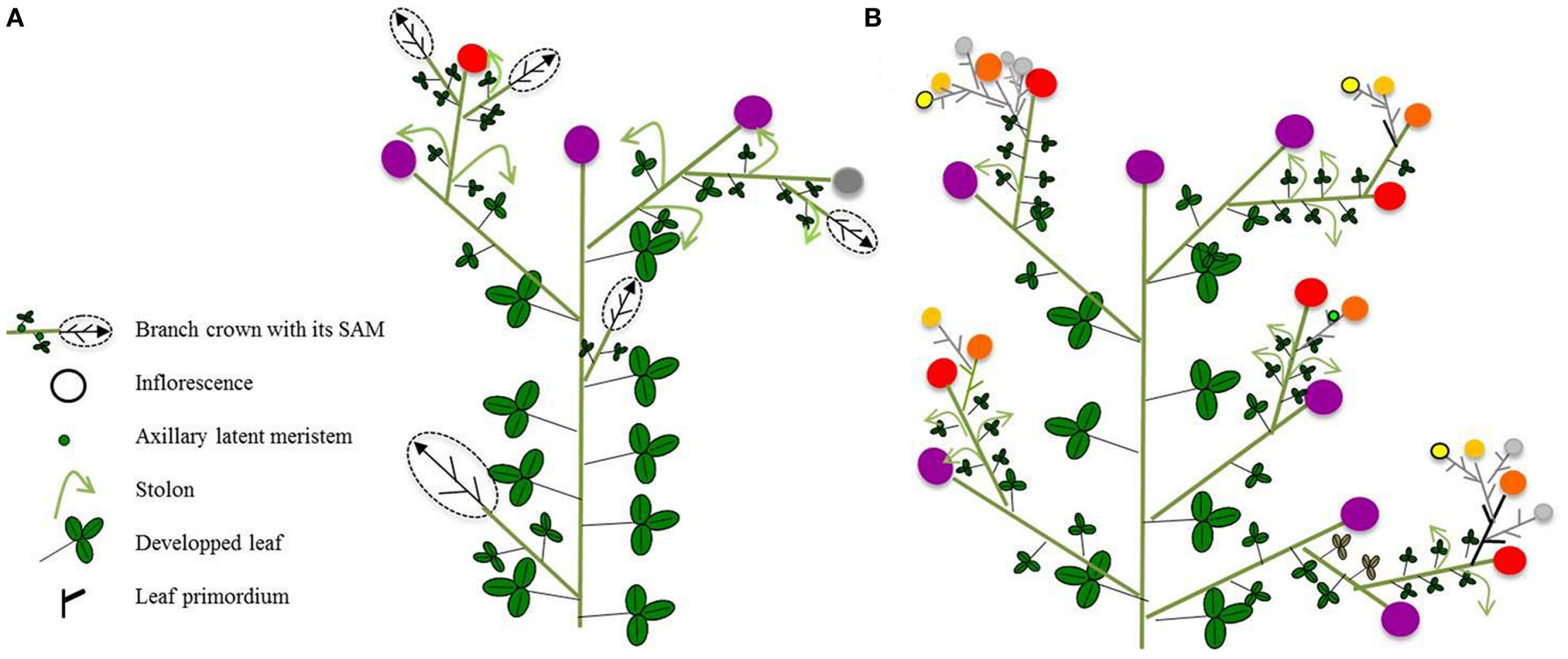
**Architecture of a strawberry plant. (A)** for a once flowering genotype, in which axillary meristems will develop into runners or branch crowns and **(B)** for a perpetual flowering genotype, in which axillary meristems will develop into new inflorescences. The color of inflorescence reflects its stage: purple when already harvested, red when fruits are mature, dark orange when fruits are developing, light orange or yellow when inflorescence newly emerged, and gray when inflorescence will likely not emerged. Small green dot represents axillary latent meristem observed in perpetual flowering genotypes.

Compared to the other Rosaceae model plants described above, strawberry is an herbaceous perennial. In strawberry, cultivated or woody, the branching is sympodial, with floral initiation occurring terminally. Extension axes can develop in the uppermost axillary buds below the terminal inflorescence (Battey et al., 1998) or in the basal parts of the primary crown (Sugiyama et al., 2004), giving birth to new crowns with terminal flowering (Figure 5A). These new crowns are usually smaller than the primary crown and thus correspond to sequential branching. In addition to sexual reproduction, new plants can develop from primary stolons which are specialized and highly elongated axes developing at the first two nodes of a mother plant. Stolons can be considered as reiteration of the entire plant with roots. As in rose, it is possible to distinguish two types of behaviors, non-recurrent (or once flowering) and recurrent (perpetual flowering) (Gaston et al., 2013). In non-recurrent genotypes, low temperature and short days in fall trigger floral initiation (Verheul et al., 2007) with autumn-initiated flowers emerging in the next spring. In contrast, in recurrent genotypes, flowers are initiated continuously during the growing season from spring until late autumn (Battey et al., 1998; Savini et al., 2005).

Whatever the flowering type, stolons emerge in long days from basal axillary buds, elongate and produce new plants through the so-called runnering process (Savini et al., 2008). Each new clonal plant is composed of a very short stem called a rosette (Darrow, 1966). In non-recurrent genotypes, lateral buds remain dormant or develop into either axillary shoots (branch crowns) or stolons depending on their positions (Guttridge, 1956) (Figure 5A). In short days, floral initiation occurs in apical meristems in which no further vegetative development will be observed. Due to apical dominance, the number of flower bud primordia increases as the stage of development of the primary flower advances (Jahn and Dana, 1970). For recurrent genotypes and as described in rose, all the axes developing during a growth season are floriferous, with terminal flowering (Figure 5B).

## Synthesis and discussion

In this review we have shown that different species are characterized by a typical organization of branching and flowering traits. How these organizations are modulated depends on internal factors such as the ontogenetic stage of the parent shoot or the genotype (see Supplementary Material—Changes in branching pattern with plant ageing (ontogeny) and Variability of branching pattern depending on the genotype, respectively). Moreover, branching patterns exhibit plasticity depending on cultural management and climatic conditions, either in fields or in greenhouses. Examples of this plasticity are provided in Supplementary material, focusing on tree management in the case of fruit trees and on the effects 47 of climatic controlled conditions in rose and strawberry.

**Figure 6:**
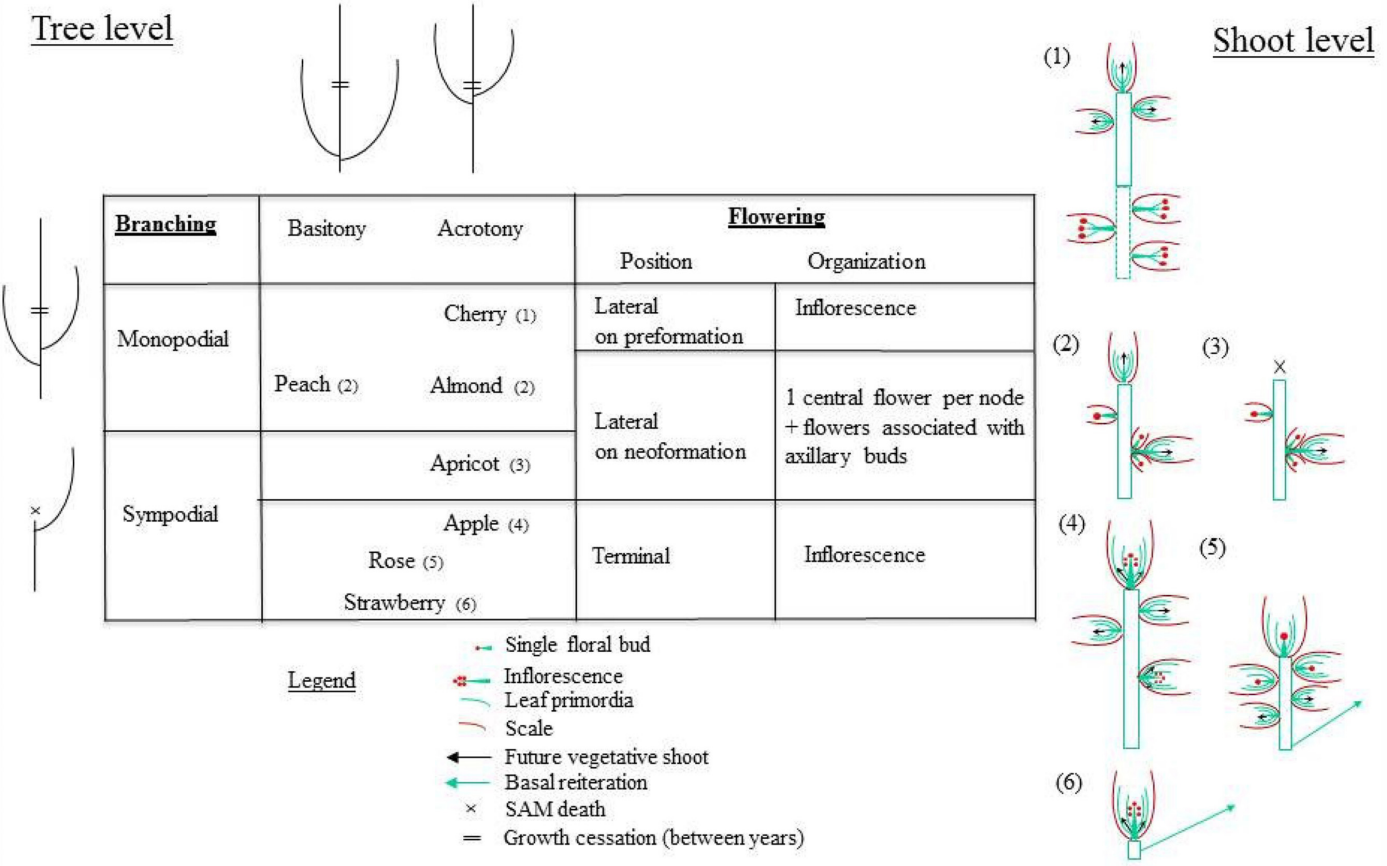
**Synthesis of the branching organization at the tree and shoot scales, in relation to vegetative and reproductive bud position and internal structure, in a range of Rosaceae species**.

A limited number of species and genera of the Rosacea family were considered which belong to two distant sub-taxa, *Rosoideae* for *Rosa* and *Fragaria, Spiroidae* for *Prunus* and *Malus* (Potter et al., 2007). Even though no variability exists in the phyllotactic angle (2/5 in all species) there is large variability in branching patterns that results from bud topology and fate configurations (Figure 6): (i) vegetative buds may develop into long shoots, in the distal or proximal zones along the parent shoots or at the plant level, and (ii) the vegetative and floral buds can be separate or mixed. Also, the timing and location of floral induction in buds differ among species. The bud and tissue configurations observed in this set of closely related species question the relationships between apical meristem maintenance, determinacy and floral induction, and the resulting morphotypes. Indeed, meristem maintenance is essential for perennials and the flowering strategy may be accounted for by differences in the regulation of meristem identity, developmental phases (juvenile vs. adult) and determinacy (Battey and Tooke, 2002).

### Morphotypes result from shoot apical meristem maintenance, determinacy and floral induction location at the shoot scale

The most forest-type morphotype, represented by the cherry example, combines a strong acrotony—at both tree and shoot level—with a systematic separation of floral and vegetative meristems. The location of flowering buds in preformed zones, far from the SAM, allows the end of the juvenile period without altering the vegetative growth capacity of the SAM. By maintaining a long organogenetic period in the SAM of orthotropic axes, especially in the main axis, a tall tree is constructed, that is able to compete for light with its neighbors in a forest habitat. In contrast, the closer the floral tissues are from the apex the higher SAM determinacy. Among *Prunus* species, which all exhibit floral and vegetative buds combined along the neoformed part of the shoots, almond has a more upright and acrotonic shape compared to peach (more basitonic) or apricot (sympodial branching), which exhibit smaller tree sizes. The periodic death of SAM without floral differentiation, as observed in apricot, is an interesting case which suggests a deficiency in SAM maintenance. It could reflect SAM sensivity to climatic conditions. Also, stopping and resuming growth frequently may allow a flexible growth and to be more adapted to dry ecosystems in which this species is grown (Kodad et al., 2013).

The proximity between floral induction and SAM is maximal in the case of terminal flowering leading to sympodial branching. This sympodial branching when associated to a basitonic branching, at least for reiteration, leads to more bushy and creeping morphotypes, as in rose and strawberry. The present review revealed that apple tree, rose, and strawberry share common characteristics due to the terminal position of flowering and mixed buds with vegetative organs (leaf and shoot primordia) and flowers or inflorescences. Strawberry represents an extreme case of reduction of the vegetative apparatus into a rosette with axillary shoots (morphologically equivalent to a bourse and bourse shoot in apple) and a strong reiteration process via runnering. The main differences between these species result from (i) the position of long shoots and reiteration, mainly acrotonic in apple, either acrotonic when branching sequentially or basitonic when reiterating in rose and strawberry; (ii) the time interval between two consecutive flowering occurrences, from pluri-annual in apple to annual in once-flowering genotypes or intra-annual in recurrent-flowering genotypes of rose and strawberry. Even though transgenic plants could be induced to perpetual flowering when *AtFT* was overexpressed in apple, vegetative growth was not maintained and the plant died (Tanaka et al., 2014). Equilibrium between vegetative and floral phases is thus required for plants to develop and survive.

Common molecular mechanisms between these species may be involved in the SAM maintenance, determinacy and flowering on the one hand and in the monopodial/sympodial and acrotony/basitony branching on the other hand. Indeed, the role of *TFL1/FT*-like genes in the shoot-identity of meristems has been demonstrated in contrast to “flowering genes” such as *LEAFY* or *APETALA* (Larsson et al., 1998; Parcy et al., 2002). In Rosaceae, homologs of *TFL1-like* genes might have diverged evolutionary in function and expression. In particular, the number of copies for the different genes differs between species (Esumi et al., 2009; Mimida et al., 2012, 2013), thus leading to divergences in the regulating networks controlling meristem identities. This could explain the variations detailed in this review. However, *TFL1* homologs have been shown to have a repressive effect on flowering in most species described in this paper (rose: Randoux et al., 2013; strawberry: Gaston et al., 2013; apple: Mimida et al., 2013). Moreover, in *Rosa* and *Fragaria*, mutations on *TFL1* lead to extreme branching behavior with all developing axes becoming floriferous (Iwata et al., 2012). This process was related to the role of orthologs of At*TFL1* in indeterminate and determinate genotypes in pea (Tian et al., 2010) and in tomato (Shalit et al., 2009). In contrast, less attention has been paid to other genes such as *WUSCHEL* (*WUS*) which are responsible for the maintenance of cell population at the SAM in model plants.

### Branching patterns result from the location axillary flowering in either preformed or neoformed parts of the parent shoot

In most species considered in this review, buds are formed and their fate determined during a growing season whereas they grow after a dormant winter period, in the following spring. This is typical of perennial structures, aside from the notable exception of sylleptic branching. Among the population of buds developed during a growing season, only a portion differentiates into flowers, at specific positions. These positions can also be viewed as particular time steps during the annual shoot development. In the cherry tree case, floral induction occurs in buds located in the preformed part of the shoot only, thus in a small number of meristems formed early during the season. This could result from the temporary expression of key genes as previously shown in poplar, in which a peak of *FT1* expression in winter initiates the transition of vegetative meristems to floral state whereas buds produced before and after *FT1* expression are vegetative (Hsu et al., 2011). In the other *Prunus* species, including peach and almond, floral differentiation also occurs in preformed organs but associated with axillary meristems that are formed all during the growing season and along the neoformed part of the parent shoot. This suggests that the conditions of floral induction must be maintained throughout the growth season. Moreover, the recurrence of branching zones associated to the number of flowers, as found in apricot or almond, suggests that floral induction occurs in different waves of intensity leading to a variable (increasing or decreasing) number of flowers associated with axillary meristems. These fluctuations could result from fluctuating environmental conditions or internal growth conditions involving resource availability or molecular controls. Even though investigations are required to clarify the contribution of these different factors, it is suspected that in such tissue configurations, the regulation of cellular territory fates must be very precise in time and local space. In particular the equilibrium between TFL1 and FT genes may be regulated very precisely, in local cell territories (Mimida et al., 2011).

### Terminal flowering and growth cessation

In species characterized by terminal flowering, the shoot apical meristem is not able to maintain its organogenic activity during an indeterminate period and floral induction occurs when this acitivity ceases. Because floral induction occurs either on short or long shoots which stop growing at different periods, the conditions of floral induction must be maintained throughout the growing season. Several authors have suggested that the constitution of an embryonic shoot in terminal floral buds in apple requires that organogenic activity is maintained until the winter rest period (Fulford, 1966b) and this could be related to the growth speed of the parent shoot (Crabbé, 1987). These findings are consistent with the location of the floral zone in the distal third of annual shoots, between the sylleptic zone which is considered as the fastest growing zone, and terminal growth cessation (Costes and Guédon, 2002). Therefore, as suggested by van der Schoot et al. (2014), growth cessation and the constitution of the embryonic shoot within the dormant bud may be a key step for perennial species with a dormancy period and terminal flowering. This is supported by the fact that floral promoting *FT*-like genes are likely to be involved in seasonal growth and dormancy in trees (Bohlenius et al., 2006; Hsu et al., 2011) and have also been shown to regulate branching in some trees (Srinivasan et al., 2012). Even though van der Schoot et al. (2014) insisted on the environmental control of meristem activity, especially during the period of growth cessation prior to dormancy, the nutritional status of each bud during this period is likely to be as important as climatic conditions. This is supported by physiological studies that emphasized the role of carbohydrates in floral induction (Bangerth, 2009; Wahl et al., 2013), as well as in axillary shoot control (Mason et al., 2014).

## Conclusion

Examining the topology and fates of meristematic and floral tissues at more global scales shows that the differentiation of tissues issuing from meristems is not limited to leaf emergence and phyllotactic arrangement but complex and precise spatial and temporal regulation operate in the populations of meristems constituting polycarpic plants. The quantification and modeling of bud fates depending on their position makes possible the simulation of plant development over time (e.g., Lopez et al., 2008). In turn, the simulation of realistic plant structures allows estimating interactions with environmental conditions and/or between organs competitions in structural-functional plant models. The spatial and temporal regulation of bud fates certainly involves the coordination of several classes of genes which control meristem organization, maintenance and identity. Part could be common to different processes involved in plant architecture; for instance, the pleiotropic effect of gibberellins on both cell division and cell elongation (Yamaguchi, 2008; Claeys et al., 2014), or TFL1 effects on meristem identity (Iwata et al., 2012; Mimida et al., 2013; Randoux et al., 2013). Finally, the factors leading to meristem fate, i.e., the length and intensity of its organogenic activity and its transition from vegetative toward floral state, are likely to involve its position relatively to other meristems, in addition to environmental factors. This may involve apical dominance and hormonal controls (cytokinins, auxin, and gibberellins), in coordination with key gene activities such as FT/TFL1-like genes (Bangerth, 2009; Mimida et al., 2011; Iwata et al., 2012; Koskela et al., 2012). But, interactions between floral differentiation and development of vegetative axillary shoots appear to be complex as suggested in a recent study showing that the branching factor *BRC1* modulates *FT* activity in the axillary buds of *Arabidopsis* (Niwa et al., 2013). Deciphering the common vs. specific molecular mechanisms driving meristem identity, determinacy and phases in several species represents a new avenue for research that will certainly benefit from revisiting plant morphology and architecture at more global scales.

### Conflict of interest statement

The authors declare that the research was conducted in the absence of any commercial or financial relationships that could be construed as a potential conflict of interest.
