# Supplementary material for: Bud structure, position and fate generate various branching patterns along shoots of closely related Rosaceae species: a review
Source: Front Plant Sci. 2014 Dec 2;5:666. doi: 10.3389/fpls.2014.00666 (PMC4251308; doi:10.3389/fpls.2014.00666)
Supplement: Supplementary file 1 [file DataSheet1.DOCX]

***Supplementary Material***

**Bud structure, position and fate generate various branching patterns along shoots of closely related Rosaceae species: a review**

**E. Costes^1*^, L. Crespel^2^, B. Denoyes^3^, P. Morel^4^, MN Démené^5^, P.E. Lauri^1^, B. Wenden^6^**

^1^INRA, UMR 1334, AGAP CIRAD-INRA-Montpellier SupAgro, «Architecture et Fonctionnement des Espèces Fruitières» Team, TA A 96/03 34398 Montpellier cedex 5, France

^2^Agrocampus Ouest, Institut de Recherche en Horticulture et Semences INRA, Agrocampus Ouest, Université d’Angers, SFR 149 QUASAV, 49045 Angers, France

^3^INRA, UMR 1332 Université de Bordeaux, INRA, Biologie du Fruit et Pathologie, F-33140 Villenave d’Ornon, France

^4^INRA, Institut de Recherche en Horticulture et Semences INRA, Agrocampus Ouest, Université d’Angers, SFR 149 QUASAV, 49071 Beaucouzé, France

^5^ Invenio, Maison Jeannette, F-24140, Douville, France

*** Correspondence:**

Dr E. Costes, INRA, UMR 1334 AGAP, Avenue Agropolis, TA A 96/03 34398

Montpellier cedex 5, France

1. **Within species variability of branching patterns**
   1. *Changes in branching pattern with plant ageing (ontogeny)*

One of the most remarkable impacts of ontogeny on plant structure is the progressive reduction of annual shoot growth with plant ageing (Nozeran, 1984). This is usually accompanied by the progressive reduction in lateral shoot length respectively to that of their parent shoot, at least in the case of sequential branching (see Barthélémy and Caraglio, 2007 for further details). All the Rosaceae species described herein exhibit such a reduction in the consecutive shoot lengths and a decrease of branching frequency. These gradients have been analysed in cherry (Lauri, 1992), apricot (Costes, 1993), apple (Costes *et al.,* 2003) with typically short shoots being the only ones developing afterward. Similarly, in rose bush, the long shoots frequency decreases with the branching order (Morel *et al.,* 2009; Crespel *et al.,* 2013). When examining the complex branching patterns at shoot level as revealed in peach and apple tree, the progressive simplification of branching appears to start with the disappearance of zones located in the middle of long shoots (Fournier *et al.,* 1998; Renton *et al.,* 2006). Indeed, sylleptic shoots, usually in mesotonic positions, have been shown to develop only on very vigorous shoots with high growth rate in peach (Génard *et al.,* 1994), apple (Costes and Guédon, 2002; Renton *et al.,* 2006) and almond (Negron *et al.,* 2014). However, sylleptic shoots are absent along epicormic shoots compared to long proleptic shoots in almond (Negron *et al.,* 2013). In apple, when the parent shoot category decreases from long to medium (which length is between 5 to 20 cm), the branching zones length and occurrence progressively decrease, with an almost linear decrease of the floral zone length (Renton *et al.,* 2006). The branching pattern of medium shoots is composed of an axillary floral zone followed by few short or medium laterals on their distal part, which corresponds to an acrotony reminiscence, and some latent buds at their bottom and top. The short shoots (or spurs) which number increases with the tree age are usually not branched but still bear a few flowers.

- 1. *Variability of branching pattern depending on the genotype*

As previously shown, each species is characterized by a typical organization of growth, branching and flowering traits. However, within each species significant differences between cultivars have been described for growth (e. g. the number of leaves per shoots as a function of tree age), branching (see below) and/or geometry. This variability can be described at different scales, from the whole plant to the shoot and organ scales. At whole plant scale, tree shapes are often considered in their global geometry and classified from upright to weeping (e.g. -Scorza 1984; Scorza *et al.,* 2002, for peach) or as spindle- or more round-shaped tree structure (e.g. Lauri and Claverie, 2008, for cherry). These classifications mainly result from visual estimation of branching angles, branch orientation, tree volume and shape. More detailed descriptions of the differences in the whole plant geometry between genotypes can be obtained by digitizing (e.g. Crespel *et al.,* 2013 for rose).

In apple, even though still at the whole tree shape, a more precise description of architectures has been proposed in which cultivars have been classified as either lateral or terminal bearer depending on whether flowering develops predominantly in lateral or in terminal buds on the long shoots (Forshey *et al.,* 1992). Lespinasse and colleagues have established a typology including four main fruiting types from type I for cultivars with fruiting on old wood (4-year old and older) to type IV for cultivars with terminal bearing (Lespinasse, 1977; Lespinasse and Delort, 1986; Hampson and Kemp, 2003). The quantitative comparison of branching patterns at more local scales, i.e. along shoots with similar length (or sampled at similar ontogenetic stages), between genotypes revealed a common organization in consecutive zones in all the genotypes. The branching zones are similar but the length and frequency of lateral types within a given zone differ between genotypes (Costes and Guédon, 2002). In cherry, the basic branching pattern with a clear separation between zones with vegetative axes and lateral flowering has also been shown to vary depending on the genotype, with sour cherry having more flower buds on long shoots than sweet cherries (Thompson, 1996). Also, the relative reduction of the lateral shoot lengths in comparison to the parent shoot contributes, with branching angles, to the spread *vs* erected shape of peach trees (e.g. Dardick *et al.,* 2013). In strawberry, as shown previously in Figure 5, branching pattern depends mainly on the flowering behavior of genotypes, once flowering vs perpetual flowering. Beside this distinction, Sugiyama *et al.* (2004) has highlighted the variation in the degree of apical dominance, resulting in different spatial and temporal fates of dormant axillary buds. In genotypes with strong apical dominance, many axillary buds remain dormant at planting and develop, at a later stage, into basal branch crowns while in genotypes with less apical dominance they will develop into basal crowns or stolons just after planting.

- 1. *Plasticity depending on cultural management and climatic conditions*

Depending on the production conditions, in field or greenhouses, the studies performed on plant plasticity have focused mainly on the effects of either management or climatic conditions, respectively. As a consequence, the plasticity of branching patterns depending on cultural management are exemplified in what follows mainly on tree species whereas plasticity to climatic conditions are illustrated in rose and strawberry.

In fruit trees, although characteristics intrinsic to each genotype are useful in themselves to adapt the pruning and training system (Lauri *et al.,* 2011), different cultivars may have various responses to a same extrinsic factor, whether climatic or related to tree management. Shoot bending or leaning is often used as cultural management. In Cherry tree, this changes the branching angle and, in turn, influences the number of inflorescences per spurs (Flore and Layne, 1996; Thompson, 1996) as well as the number of flowers per spur and fruit-set (Lauri *et al.,* 1998). Also, in apple tree, shoot bending may significantly increase flowering on one-year-old wood compared to the upright branch, with however differences between the cultivars (Lauri and Lespinasse, 2001).

Pruning also provokes contrasted responses in branching and flowering along the unpruned part of the shoot depending on whether pruning is done in winter or during the growing season (Fumey *et al.,* 2011). In cherry, summer pruning is likely to modify the time of initiation of flower buds by 1- to 2-weeks compared to floral buds borne on unpruned shoots or spurs, this reaction being linked to limitations in developmental resources (Guimond *et al.,* 1998). Also, rootstocks are widely used to control the fruit tree aerial development. Their influence on branching pattern has been described in apple tree by comparing different rootstock and inter-stock combinations on the one-year-old scion branching zonation (Seleznyova *et al.,* 2007). This study shows that dwarfing rootstocks increase the frequency of flowering, and the length and density of the axillary flowering zone.

In orchards conditions, trees are in most cases irrigated. Even though a large number of studies have been dedicated to the effect of irrigation timing and reduction on fruit production (Naor *et al.,* 2008 in apple; Lopez *et al.,* 2006 in peach), studies related to the effects on shoot morphology and branching remain scarce. The effect of water deficit on shoot growth rate and branching pattern has been recently studied in Almond, cv ‘Non Pareil’, and for two shoot types (long proleptic and epicormic shoots) (Negron *et al.,* 2014). Water deficit reduces growth rate in both shoot types, although patterns over the season were similar. It affected the occurrence and lengths of certain zones, but differently depending on the shoot type. In proleptic shoots, only the first basal zones were impacted. In contrast, in epicormic shoots, the median zone was more frequently skipped even though the total number of nodes per shoot was not reduced by water deficit. Moreover water deficit had a minor effect on the zone flowering and did not modify shoot or zone composition of axillary meristem fates. Finally, water deficit impacts the structure of proleptic shoots more than that of epicormic shoots, consistently with their different ontogenetic characteristics and/or resource availability for development. This study suggests that water deficit could stimulate a more rapid progression through ontogenetic states, i.e. accelerate tree ageing.

As for water stress, few studies have been performed on the effect of light or temperatures on branching due to the difficulty to control these factors. Studies mainly focused on the effects of light transmission through nets of different colours on carbon assimilation and fruit production (e.g. Bastias and Corelli-Grapadelli, 2012). Also, a large bibliography is available on the effects of temperatures, from fall to spring, to fulfil chilling and heating requirements and on the timing of bud-break (e.g. Campoy *et al.,* 2011). A recent study shows clear effects of winter temperature on dormancy completion for which winter temperature has a pivotal role in the frequency and phenology of budburst but with a consistent effect of the cultivar on the phenology of lateral outgrowth (Dutra-Schmitz *et al*. 2014).

Regarding the main environmental factors that can be controlled in greenhouse (light, temperature and water), the rose demonstrates a great architectural plasticity. In particular bud burst and the number of floriferous axes may greatly vary as well as their length. The light is one of the main factors which affect plant architecture in rose. Indeed, the light is absolutely necessary to trigger bud burst in rose, the bud and not the stem being the active site for light perception (Girault *et al.,* 2008). A reduction in light intensity reduces the frequency of budburst, and of floriferous axes. Indeed, the reduction of 10, 35, 60 and 70% of light intensity by shading the plants under greenhouse during winter, reduces the number of axes with flowers per plant of 43, 65, 75 and 90% respectively (Zieslin and Halevy, 1969). In contrast, increasing the cumulative irradiance of 23.5% between two successive cultures, led to an increase in the number of floriferous axes by 173% on average (Crespel *et al.,* 2014). An average increase in the length of these axes of 7.5% was also observed, with different patterns of responses, even opposed, depending on the cultivar. Similar observations have been obtained for roses grown with supplemental lighting (Carpenter and Rodriguez, 1971). A low light intensity (91 µmol.m-2.s-1) applied to young plants, grown in culture chamber, for 16 days followed by a high light intensity (580 µmol.m-2.s-1), led to an increase in the number of floriferous axes of 35% (Demotes-Mainard *et al.*, 2013). As regards the quality of the light, the relationship between the wavelengths also affects the architecture of rose plants. A high red to far red ratio (R/FR) increased the frequency of bud burst (Healy and Wilkins, 1979), even though no effect of R/FR on bud burst at bud level (Wubs *et al.,* 2014) and no impact on the height of the plant (Cerny *et al.,* 2003) were found. Also, a complement of blue light brought by LED made it possible to reduce of 14.2% the length of axes compared to lighting with sodium-vapor (Terfa *et al.,* 2012). However, blue lighting did not modify the architecture of the plants compared to a white lighting, in culture chamber conditions (Abidi *et al.,* 2012).

The temperature has been shown to be the second factor after light which most affects the rose plant architecture. For a constant night temperature of 16°C, an increase in the day temperature from 21 to 32°C led to an increase in the number of bud burst and thus floriferous axes of 84.6%, and a reduction their length of 60.7% (Byrne *et al.,* 1978). Also in young rose plants, grown in greenhouse that were subjected to water restriction for 7 to 35 days, followed by a return to water comfort, an increase in the number of bud burst (41 to 54%) was observed. However, the number of floriferous axes did not increase because of more numerous aborted (or “blind”) axes (Demotes-Mainard *et al.,* 2013).

In strawberry, flower induction is mainly controlled by an interaction between temperature and photoperiod (review in Heide *et al.,* 2013), which also controls the formation of stolons (Battey *et al.,* 1998). In addition, genotype effect can modulate the flowering quantitative response offering a large variation in these traits (Opstad *et al.,* 2011). Usually, short days (SD) with about 15°C are required for flowering in once-flowering genotypes and runnering takes place in long days (LD). However, in SD, below 15°C and above 9°C, plants flowers whatever the photoperiod, while high temperatures delay or even prevent floral initiation (Taylor, 2002). This situation leads to a variation of the architectural pattern in achieving floral initiation in a single genotype cultivated in same conditions but in different years or different latitudes.

Other environmental factors regulating the flowering and therefore the architecture of the plant are the quality or intensity of light and nutrients. Daylength extension with light spectral bands limits floral initiation when performed near midnight (extension with R light) or in the evening (extension with FR light) (Vince-Prue and Guttridge, 1973). High light intensity, e.g. 400 vs 200 micromol m^-2^ s^-1^, favours vegetative propagation through the formation of stolons (Wu *et al.,* 1999), and limits floral initiation (Démene pers. observation). Also, low level of nutrients such as nitrogen during induction get earlier floral initiation (Sonsteby *et al.,* 2009), while nitrogen fertilization after beginning of SD exposure enhances floral initiation in once flowering strawberries (Opstad *et al.* 2011). Chilling requirement is also an important factor for a balanced vegetative and floral development (Sonsteby *et al.* 2006). When chilling requirements are not satisfied, e.g. 400 hours below 7°C instead of 800 for ‘Gariguette’ cv, the plants are stunted after dormancy, inflorescences emerged more rapidly and the ratio flowers/leaves is modified. To solve this problem, exposures to short flash light are performed.

In conclusion, branching intensity, phenology and patterns exhibit a large plasticity in response to cultural and climatic conditions. In the future, more studies will certainly be required to estimate the relative variation of genotypes, environmental factors and their interaction in Rosaceae species. So far few studies have been performed in this domain, maybe due to the difficulty to perform dedicated experimentations, especially for trees. Moreover, deciphering the effects of ontogeny, climatic conditions and other fixed factors from genetic effects on observed phenotypes may require complex modelling approaches in which dependencies between developmental stages and/or axes within the plant structure must be taken into account (see e.g. Segura et al., 2008). However, the plasticity described in this section does not suppress nor invalidate the intrinsic organisation of each species, as described in the main paper.

1. **Supplementary Figures and Tables**

## Suplementary Tables: Supplementary Table 1.

**Glossary of terms (***Definition mainly from Barthélémy and Caraglio (2007) which can be seen for details and illustrated examples).*

Apical dominance is the control exerted by the growing terminal bud, including the apical meristem, over the outgrowth of axillary meristems (Cline, 1997) whereas apical control is the suppression of growth in lower branches by a higher dominant branch(es) or leader shoot(s) (Cline *et al.* 2009).

Organogenesis (or organogenetic activity) is the inception of new organs which results from the functioning of the undifferentiated cells that constitute the shoot apical meristem.

Continuous flowering or perpetual flowering: Capacity to develop floral meristems in the SAM all along the growth period.

Immediate versus delayed branching: Once initiated, an axillary meristem may remain dormant or can develop into a lateral axis. A lateral axis may elongate immediately after axillary meristem initiation or after a phase during which the axillary meristem remains inactive, very often protected in a bud. These different timing in lateral development are referred to as immediate or delayed branching, respectively(i.e. ‘sylleptic’ or ‘proleptic’, sensu Hallé *et al.,* 1978).

Monopodial versus sympodial branching: Depending on the indeterminate or determinate growth of an axis, its branching is monopodial or sympodial, respectively. In the latter case, one, two or more branches may develop after the death or transformation of the SAM.

Acrotony is the preferred development of lateral axes in the distal part of a parent axis or shoot;

Mesotony corresponds to a privileged development of branches in the median part of a shoot or axis.

Basitony is the privileged development of lateral axes in the basal part of a vertical stem or shoot. This may involve the whole plant, the axis or the growth unit level only.

Growth unit: portion of an axis which develops during an uninterrupted period of extension

Preformed/Preformation: metamers and organs of a future elongated shoot that are at an embryonic stage in a bud before shoot elongation

Neoformed/Neoformation: organs that were not included at an embryonic stage in a bud but formed during the morphogenesis and elongation period of the shoot

Sequential branching: sequence of axes which number of categories is finite and corresponds to the architectural unit of each species. Numbering of branching orders start with the main axis (order 0) and increase towards the ultimate shoot.

Reiteration: Process during which a plant duplicates its own elementary architecture, i.e. its architectural unit. Reiteration may be total or partial and encompasses several aspects such as sprouts, root-suckers, reaction to pruning, etc.

1. **Supplementary References**

Abidi, F., Girault, T., Douillet, O., Guillemain, G., Sintes, G., Laffaire, M., Ben Ahmed, H., Smitin S., Huché-Thélier, L., Leduc, N. (2012). Blue light effects on rose photosynthesis and photomorphogenesis. *Plant biology* 15, 67-74.

Barthelemy, D., Caraglio, Y. (2007). Plant Architecture: A Dynamic, Multilevel and Comprehensive Approach to Plant Form, Structure and Ontogeny. *Ann. Bot.* 99, 375–407.

Battey, N.H., Le Mière, P., Tehranifar, A., Cekic, C., Taylor, S., et al. (1998). “Genetic and environmental control of flowering in strawberry” in Genetic and environmental manipulation of horticultural crops, 111-131.

Bastías, R. M., Corelli-Grappadelli, L. (2012). Light Quality Management in Fruit Orchards: Physiological and Technological Aspects. *Chile. J. Agr. Res.* 72, 574–81.

Byrne, T.G., Doss, R.P., Tse, A.T.Y. (1978). Flower and shoot development in the greenhouse roses, ‘Cara Mia’ and ‘Town Crier’under several temperature-photoperiodic regimes. *J. Am. Soc. Hort. Sci.* 103, 500-502.

Campoy, J.A., Ruiz, D., Egea, J. (2011). Dormancy in temperate fruit trees in a global warming context: a review. *Sci. Hort.* 130, 357-372

Carpenter, W.J., Rodriguez, R.C. (1971). Supplemental lighting effects on newly planted and cut-back roses. *HortScience* 6: 207-208.

Cerny, T.A., Faust, J.E., Layne, D.R., Rajapakse, N.C. (2003). Influence of photoselective films and growing season on stem growth and flowering of six plant species. *J. Am. Soc. Hort. Sci.* 128, 486-491.

Costes, E. (1993). Architecture aérienne de l'abricotier en conditions naturelles. Acta Botanica Gallica, 140, 249-261.

Costes, E., Guédon Y. (2002). Modelling branching pattern on one-year-old trunks of apple cultivars. *Ann. Bot.* 89, 513-523.

Costes, E., Sinoquet, H., Kelner, J.J., Godin, C. (2003). Exploring within-tree architectural development of two apple cultivars over 6 years. *Ann. Bot.* 91, 91-104.

Crespel, L., Sigogne, M., Donès, N., Relion, D., Morel, P. (2013). Identification of relevant morphological, topological and geometrical variables to characterize the architecture of rose bushes in relation to shape. *Euphytica* 191, 129-140.

Crespel, L., Le Bras, C., Relion, D., Morel P. (2014). Genotype x year interaction and broad-sense heritability of architectural characteristics in rose bush. *Plant breeding*. doi: 10.1111/pbr.12157.

Dardick, C., Callahan, A., Horn, R., Ruiz, K.B., Zhebentyayeva, T., Hollender C., et al. (2013). PpeTAC1 promotes the horizontal growth of branches in peach trees and is a member of a functionally conserved gene family found in diverse plants species. *Plant J.* 75, 618–30.

Demotes-Mainard, S., Huché-Thelier, L., Morel, P., Boumaza, R., Guerin, V., Sakr, S. (2013). Temporary water restriction or light intensity limitation promotes branching in rose bush. *Sci. Hort.* 150, 432-440.

Dutraz-Schmitz, J., Guédon, Y., Herter, F. G., Leite, G. B., Lauri, P.É. (2014). Exploring Bud Dormancy Completion with a Combined Architectural and Phenological Analysis: The Case of Apple Trees in Contrasting Winter Temperature Conditions. *Am. J. Bot.* 101, 398–407.

Flore, J.A., Layne, D.R. (1996). "Prunus" in *Photoassimilates distribution in plants and crops. Source-sink relationships*. eds Zamski, E., Schaffer, A.A., Marcel Dekker, Inc., New York, Basel, Honk Kong, 825-849.

Forshey, C. G., Elfving, D. C., Stebbins, R. L. (1992). *Training and pruning apple and pear trees*. Alexandria (Virginia, USA). American Society for Horticultural Science.

Fournier, D., Y. Guédon, Costes, E. 1998. A comparison of different fruiting shoots of peach trees. IVth International Peach Symposium. Bordeaux (France). *Acta Hort.* 465, 557-65

Fumey, D., Lauri, P.É., Guédon, Y., Godin, C., Costes, E. (2011). How young trees cope with the removal of whole or part of shoots: an analysis of local and distant reactions to pruning in 1-year-old apple trees. *Amer. J. Bot.* 98, 1737-1751

Génard, M., Pagès L., Kervella, J. (1994). Relationship between sylleptic branching and components of parent shoot development in the peach tree. *Ann. Bot.* 74, 465-70.

Girault, T., Bergougnoux, V., Combes, D., Viemont, J.D., Leduc, N. (2008). Light controls shoot meristem organogenic activity and leaf primordia growth during bud burst in *Rosa* sp. *Plant Cell Env.* 31, 1534-1544.

Guimond, C.M., Lang, G.A., Andrews, P.K. (1998). Timing and severity of summer pruning affects flower initiation and shoot regrowth in sweet cherry. HortScience 33, 647-649.

Hampson, C. R., Kemp, H. (2003). "Characteristics of important commercial apple cultivars." in *Apples: botany, production and uses.* eds Ferree, D. C. and Warrington, I. J., 61-89.

Healy, H.W., Wilkins, H.F. (1979). The effect of light quality on rose shoot production. *HortScience* 14, 409.

Heide, O.M., Stavang, J.A., Sonsteby, A. (2013). Physiology and genetics of flowering in cultivated and wild strawberries - a review. *J. Hort. Sci. & Biotech*. 88, 1-18

Lauri, P.É. (1992). Données sur le contexte végétatif lié à la floraison chez le cerisier (Prunus avium L.). *Canad. J. Bot.* 70, 1848 - 1859.

Lauri, P.É., Claverie J. (2008). Sweet Cherry Tree Architecture, Physiology and Management: Towards an Integrated View. *Acta Hort.* 795, 605-614.

Lauri, P.É., Claverie, J., Lespinasse, JM. (1998). The effects of bending on the growth and fruit production of sweet cherry spurs. *Acta Hort.* 468, 411-417

Lauri, P. É., Lespinasse, J. M. (2001). Genotype of apple trees affects growth and fruiting responses to shoot bending at various times of year. *J. Amer. Soc. Hort. Sci.* 126, 169-74.

Lauri, P.É., Hucbourg, B., Ramonguilhem, M., Méry, D. (2011). An Architectural-Based Tree Training and Pruning – Identification of Key Features in the Apple. *Acta Hort.* 903, 589-596.

Lespinasse, J. M. (1977). *La conduite du Pommier. I - Types de fructification. Incidence sur la conduite de l'arbre.* I.N.V.U.F.L.E.C. Paris.

Lespinasse, J. M., F. Delort. (1986). Apple tree management in vertical axis: appraisal after ten years of experiments. *Acta Hort.* 160, 120-55.

Lopez, G., Mata, M., Arbones, A., Solans, J.R., Girona, J., Marsal, J. (2006). Mitigation of Effects of Extreme Drought during Stage III of Peach Fruit Development by Summer Pruning and Fruit Thinning. *Tree Physiology* 26, 469–77.

Morel, P., Galopin, G., Donès, N. (2009). Using architectural analysis to compare the shape of two hybrid tea rose genotypes. Sci. Hort. 120, 391-398.

Naor, A., S. Naschitz, M. Peres, Gal, Y. (2008). Responses of Apple Fruit Size to Tree Water Status and Crop Load. *Tree Physiology* 28, 1255–61.

Negron, C., Contador, L. Lampinen, BD., Metcalf, SG., DeJong, T.M., Guedon, Y., Costes, E. (2013). Systematic Analysis of Branching Patterns of Three Almond Cultivars with Different Tree Architectures. *J. Amer. Soc. Hort. Sci.* 138, 407-415.

Negron, C., Contador, M.L, Lampinen, B.D, Metcalf, S.G., Guédon, Y., Costes, E., DeJong T.M. (2014). Differences in proleptic and epicormic shoot structures in relation to water deficit and growth rate in almond tree (*Prunus dulcis*). *Ann. Bot.* 113, 545-554.

Nozeran, R. 1984. "Integration of organismal development" in *Positional controls in plant development*. eds P.W. Barlow and D.J. Carr 375-401.

Opstad, N., Sonsteby, A., Myrheim, U., Heide, O.M. (2011). Seasonal timing of floral initiation in strawberry: Effects of cultivar and geographic location. *Sci. Hort.* 129, 127-34

Renton, M., Guédon, Y., Godin, C., Costes, E. (2006). Similarities and gradients in growth-unit branching patterns during ontogeny in ‘Fuji’ apple trees: A stochastic approach. *J. Exp. Bot.* 57, 3131-3143.

Scorza, R. (1984). Characterization of four distinct peach tree growth types. *J. Am. Soc. Hort. Sci.* 109, 455-457.

Scorza, R., Bassi, D., Liverani, A. (2002). Genetic interactions of pillar (columnar), compact, and dwarf peach tree genotypes. *J. Amer. Soc. Hort. Sci.* 127, 254-261.

Segura, V., Cilas, C., Costes, E. (2008). Dissecting apple tree architecture into genetic, ontogenetic and environmental effects. I: Mixed linear modelling of repeated spatial and temporal measures. *New Phytol.* 178, 302-314.

Seleznyova, A., White, M, Tustin, S., Costes, E. (2007). Analysis of the Earliest Observed Expression of Dwarfing Rootstock Effects on Young Apple Trees, Using Markovian Models. *Acta Hort*. 732, 79-84.

Sonsteby, A., Heide, A.M. (2006).Dormancy relations and flowering of the strawberry cultivars Korona and Elsanta as influenced by photoperiod and temperature. *Sci. Hort.* 110: 57-67

Sonsteby, A., Opstad, N., Myrheim, U., Heide, A.M. (2009). Interaction of short day and timing of nitrogen fertilization on growth and flowering of 'Korona' strawberry (Fragaria x ananassa Duch.). *Sci. Hort.* 123, 204-09

Sugiyama, N., Iwama, T., Inaba, Y., Kurokura, T., Neri, D. (2004). Varietal differences in the formation of branch crowns in strawberry plants. *J. Japan. Soc. Hort. Sci.* 73, 216-20.

Taylor, D.R. (2002). "The physiology of flowering in strawberry" in Proceedings of the Fourth International Strawberry Symposium, Vols 1 and 2, eds. Hietaranta, T., Linna, M.M., Palonen, P. Parikka, P, 245-51.

Terfa, M.T., Poudel, M.S., Roro, A.G., Gislerød, H.R., Olsen, J.E., Torre, S. (2012). Light emitting diodes with a high proportion of blue light affects external and internal quality parameters of pot roses differently than high pressure sodium lamps. *Acta Hort.* 956, 635-642.

Thompson, M. (1996). "Flowering, pollination and fruit set" in *Cherries: Crop Physiology, Productions and Uses*, eds Webster, A.D., Looney, N.E., CAB Intl., Oxon, UK. 223-241.

Vince-Prue, D., Guttridge, C.G. (1973). Floral initiation in strawberry: Spectral evidence for the regulation of flowering by long-day inhibition. *Planta* 110, 165-72

Wu, C.C., Hsu S.T., Chang M.Y., Fang W. (2011) Effect of Light Environment on Runner Plant Propagation of Strawberry. In: Goto E, Hikosaka S (eds) Vi International Symposium on Light in Horticulture. *Acta Hort.* 907, 297-302.

Wubs, A.M., Heuvelink, E., Marcelis L.F.M., Buck-Sorlin G.H., Vos, J. (2014). Axillary budbreak in a cut rose crop as influenced by light intensity and red:far-red ratio at bud level. *J. Amer. Soc. Hort.* Sci. 139, 131-138.

Zieslin, N., Halevy, A.M. (1969) The ‘blindness’ phenomenon in ‘Baccara’ rose. *Annu. Rep. Dep. Flor.* Hebrew Univ, Jerusalem, 6-8.
